# Bcl-xL Affects Group A *Streptococcus*-Induced Autophagy Directly, by Inhibiting Fusion between Autophagosomes and Lysosomes, and Indirectly, by Inhibiting Bacterial Internalization via Interaction with Beclin 1-UVRAG

**DOI:** 10.1371/journal.pone.0170138

**Published:** 2017-01-13

**Authors:** Shintaro Nakajima, Chihiro Aikawa, Takashi Nozawa, Atsuko Minowa-Nozawa, Hirotaka Toh, Ichiro Nakagawa

**Affiliations:** Department of Microbiology, Graduate School of Medicine, Kyoto University, Yoshida-Konoe-cho, Sakyo-ku, Kyoto, Japan; Univerzitet u Beogradu, SERBIA

## Abstract

Anti-apoptotic Bcl-2 and Bcl-xL are proposed to regulate starvation-induced autophagy by directly interacting with Beclin 1. Beclin 1 is also thought to be involved in multiple vesicle trafficking pathways such as endocytosis by binding to Atg14L and UVRAG. However, how the interaction of Bcl-2 family proteins and Beclin 1 regulates anti-bacterial autophagy (xenophagy) is still unclear. In this study, we analyzed these interactions using Group A *Streptococcus* (GAS; *Streptococcus pyogenes*) infection as a model. GAS is internalized into epithelial cells through endocytosis, while the intracellular fate of GAS is degradation by autophagy. Here, we found that Bcl-xL but not Bcl-2 regulates GAS-induced autophagy. Autophagosome-lysosome fusion and the internalization process during GAS infection were promoted in Bcl-xL knockout cells. In addition, knockout of Beclin 1 phenocopied the internalization defect of GAS. Furthermore, UVRAG interacts not only with Beclin 1 but also with Bcl-xL, and overexpression of UVRAG partially rescued the internalization defect of Beclin 1 knockout cells during GAS infection. Thus, our results indicate that Bcl-xL inhibits GAS-induced autophagy directly by suppressing autophagosome-lysosome fusion and indirectly by suppressing GAS internalization via interaction with Beclin 1-UVRAG.

## Introduction

Group A *Streptococcus* (GAS; *Streptococcus pyogenes*), a clinically important bacterial pathogen, causes mild and severe human diseases [1]. GAS can adhere to and invade epithelial cells through endocytosis. As a result, host cells have developed the several molecular mechanisms to eliminate the invading bacterium such as autophagy, an intracellular degradation system and apoptosis for removing infected cells themselves. For example, apoptosis in response to GAS internalization is triggered by actin rearrangement and small GTPase Rac1-mediated ROS production and prevents the spread of GAS infection through programmed suicide [2, 3], and this apoptotic cell death is inhibited by the overexpression of Bcl-2 [2]. In addition, after internalization, GAS escapes from endosomes into the cytoplasm by disrupting the endosomal membrane by way of the secreted hemolytic enzyme streptolysin O (SLO) [4], however, cytoplasmic GAS is targeted and destroyed by autophagy [5]. GAS strains are classified under serotypes associated with human disease. Strain JRS4 is a serotype M6 clone of GAS and is degraded by autophagy efficiently, so this infection model is widely used to prove the induction mechanism of GAS-induced selective autophagy. However, GAS JRS4 is not representative of all GAS strains because it is much less prevalent associated with human disease [6]. Recently, Barnett et al. reported that the globally disseminated serotype M1T1 clone of GAS (strain 5448) can avoid autophagy pathway [7].

Anti-apoptotic Bcl-2 family members such as Bcl-2 and Bcl-xL attenuate apoptosis either by sequestering proforms of death-driving cysteine proteases, called caspases, or by preventing the release of mitochondrial apoptogenic factors including calcium, cytochrome c, and AIF (apoptosis-inducing factor) into the cytoplasm [8]. In addition to anti-apoptotic function, accumulating evidence has demonstrated the multiple roles of Bcl-2 and Bcl-xL by showing their effects on intracellular trafficking including on endocytic and autophagic processes. These proteins are involved in vesicle trafficking by interacting with various trafficking related proteins including calnexin, Rab7, and vesicle associated membrane protein (VAMP) 3 [9], and in autophagy by reducing the pro-autophagic activity of Beclin 1 [10, 11].

Autophagy, a highly conserved intracellular protein-degradation system required for cellular homeostasis, provides an important intracellular immune system against invading bacteria such as *Salmonella enterica* serovar Typhimurium (*S*. *typhimurium*) [12], *Listeria monocytogenes* [13, 14], and *Shigella flexneri* [15]. These anti-bacterial autophagic responses are called xenophagy. Although anti-bacterial autophagy has common fundamental machinery with starvation-induced autophagy, such as the dependence on Atg5 and LC3 (mammalian homolog of Atg8 in yeast), its regulation mechanisms may be largely distinct from starvation-induced autophagy.

Beclin 1, the mammalian ortholog of Atg6/vacuolar sorting protein (Vps) 30 in yeast, is a Bcl-2-homology (BH)-3 domain only protein and was first discovered as a Bcl-2-interacting protein [16]. Beclin 1 associates with the class III type phosphatidylinositol 3- kinase (PI3KC3)/Vps34. This PI3KC3 produces a pool of phosphatidylinositol 3-phosphate (PtdIns3P) at the endoplasmic reticulum (ER), which is crucial for the nucleation of autophagosome formation [17]. While Bcl-2 and Bcl-xL inhibit the autophagic response by suppressing the activity of Beclin 1 [10, 11], other Beclin 1 binding molecules including the activating molecule in Beclin 1-regulated autophagy (Ambra1), UV radiation resistance-associated gene protein (UVRAG), and Atg14L, positively stimulate starvation-induced autophagy [18–21]. In addition, the UVRAG/Vps38-associating Beclin 1-PI3KC3 complex is also thought to be involved in multiple vesicle trafficking pathways including endocytosis and vacuolar protein sorting as well as autophagy [22–25].

Although possible or definite involvement of Bcl-2 and/or the Bcl-xL-Beclin 1 complex in starvation-induced autophagy and endocytic process has been demonstrated, its precise roles in internalization and autophagy during bacterial infection remain unclear. In this paper, we show the involvement of Bcl-xL, Beclin 1, and UVRAG in GAS-induced autophagy. Knockout of Bcl-xL but not Bcl-2 promotes the uptake of GAS by endocytosis, consequently leading to the promotion of GAS containing autophagosome-like vesicle (GcAV) formation. Of note, endogenous Bcl-xL also regulates autophagosome-lysosome fusion during GAS infection. Knockout of Beclin 1 also shows inhibitory function in the internalization of GAS. UVRAG can bind not only to Beclin 1 but also to Bcl-xL, and overexpression of UVRAG partially rescues the defective internalization of GAS in Beclin 1 knockout (KO) cells.

## Materials and Methods

### Cell culture and transfection

HeLa and HEK293T cell lines were purchased from the American Type Culture Collection and cultured in Dulbecco's modified Eagle's medium (DMEM, Nacalai Tesque) supplemented with 10% fetal bovine serum (Gibco) and 50 μg/mL gentamicin (Nacalai Tesque) in a 5% CO_2_ incubator at 37°C. To induce starvation, cells were incubated in Hanks’ balanced salt solution (HBSS(-), Nacalai Tesque) without serum for 2 h. Plasmid transfections were performed using polyethylenimine (Polyscience), Lipofectamine 3000 (Invitrogen), or Xfect (Clontech), according to the manufacturers’ protocols.

### Group A *Streptococcus* strain

Group A *Streptococcus* (GAS) strain JRS4 (M6^+^ F1^+^) was grown in Todd–Hewitt broth (BD Diagnostic Systems, Sparks, MD) supplemented with 0.2% yeast extract (THY), as described previously [5].

### Plasmid construction

Gateway cloning technology (Invitrogen) was used to create the vectors indicated below. Human Bcl-2 (GenBank Accession No. NM_000633.2), Bcl-xL (GenBank Accession No. NM_138578.2), Beclin 1 (GenBank Accession No. NM_003766.4), Atg14 (GenBank Accession No. NM_014924.4), and UVRAG protein cDNA (GenBank Accession No. NM_003369.3) were amplified by PCR from human cDNA libraries using the following primer pairs: Bcl-2_F, 5′-CACCATGGCGCACGCTGGGAGAACAGGGTACGAT-3′, and Bcl-2_R, 5′-TGACTTCACTTGTGGCCCAGATAGGCACCC -3′; Bcl-xL_F, 5′-CACCATGTCTCAGAGCAACCGGGAGCTGGTGGTT-3′, and Bcl-xL_R, 5′-TGGTCATTTCCGACTGAAGAGTGAGCCCAG-3′; Beclin 1_F, 5′-CACCATGGAAGGGTCTAAGACGTCCAACAACAGC-3′, and Beclin 1_R, 5′-TCATTTGTTATAAAATTGTGAGGACACCCA-3′; Atg14_F, 5′-CACCATGGCGTCTCCCAGTGGGAAGGGAGCCCGG-3′, and Atg14_R, 5′-TTAACGGTGTCCAGTGTAAGCTTTAAACCA-3′; UVRAG_F, 5′-CACCATGAGCGCCTCCGCGTCGGTCGGGGGCCCC-3′, and UVRAG_R, 5′-TCACTTATCGGAACTCCTGCGCGGCCGGCG-3′. These PCR products were cloned into the pENTR/D-TOPO vector using the pENTR Directional TOPO Cloning Kit (Invitrogen) and subcloned into the pcDNA-6.2/N-3xFLAG-DEST, pcDNA6.2/N-EmGFP-DEST, and pcDNA6.2/N-mCherry-DEST vectors. Small interfering RNAs (siRNAs) against Atg14 (5′-CCACUGCAUACCCUCAGGAAUCUAA-3′; stealth RNAi^™^ siRNA, Invitrogen) and non-specific scrambled siRNA (Invitrogen) were used for silencing experiments.

### Antibodies and other reagents

The following antibodies were used: rabbit polyclonal anti-Bcl-xL (54H6) (Cell Signaling Technology, 2764), rabbit polyclonal anti-Atg14 (Sigma-Aldrich, A6358), mouse monoclonal anti-α-tubulin (Sigma-Aldrich, T6199), mouse monoclonal anti-FLAG M2 (Sigma-Aldrich, F1804), mouse monoclonal anti-GFP (GF200) (Nacalai Tesque, 04363–24), rabbit polyclonal anti-LC3B (Sigma-Aldrich, L7543), mouse monoclonal anti-LAMP1 (Santa Cruz, sc-20011), rabbit polyclonal anti-Beclin 1 (Cell Signaling Technology, 3738), rabbit polyclonal anti-Atg5 (D1G9) (Cell Signaling Technology, 8540), rabbit polyclonal anti-Atg7 (D12B11) (Cell Signaling Technology, 8558), and mouse monoclonal anti-Galectin-3 (BD Biosciences, 556904). The secondary antibodies used for immunoblotting were horseradish peroxidase-conjugated anti-mouse or anti-rabbit IgG (Jackson Immunoresearch Laboratories). The fluorescent secondary antibodies used for immunofluorescence were Alexa Fluor 488-conjugated goat anti-rabbit IgG (Molecular Probes/Invitrogen), Alexa Fluor 568-conjugated goat anti-mouse IgG (Molecular Probes/Invitrogen), or Alexa Fluor 594-conjugated goat anti-mouse IgG (Molecular Probes/Invitrogen).

### Bacterial infection

Infections with GAS were performed as described previously [5]. GAS grown through mid-log phase was added to cell cultures at a multiplicity of infection (MOI) of 100, without antibiotics. After 1 h, infected cells were washed with phosphate-buffered saline (PBS), and then 10% DMEM/FBS with antibiotics (100 μg/mL gentamicin) was added for an appropriate period to eliminate extracellular bacteria. The cells were further cultured for the indicated times.

### Fluorescence microscopy

For immunostaining experiments, cells were washed with PBS, fixed with 4% paraformaldehyde in PBS for 15 min, permeabilized with 0.1% Triton X-100 in PBS for 5 min, and then incubated in skim milk blocking buffer (5% skim milk, 2.5% goat serum, 2.5% donkey serum, and 0.02% sodium azide in PBS containing 0.1% gelatin) or BSA blocking solution (2% BSA and 0.02% sodium azide in PBS) at room temperature for 1 h. Subsequently, the cells were incubated with primary antibodies diluted with blocking solution at 4°C overnight, washed with PBS, and then probed with secondary antibodies. Bacterial and cellular DNAs were stained with 4′,6-diamidino-2-phenylindole (DAPI) (Dojindo) in PBS. All fluorescence confocal microscopy images shown here were acquired with an FV1000 laser-scanning microscope (Olympus).

### Bacterial viability assays

Cells were cultured in 24-well culture plates and infected as described in “Bacterial infection.” After an appropriate incubation time, infected cells were washed with PBS. Infected cells were lysed in distilled water, serial dilutions of the lysates were plated on THY agar (3% Todd Hewitt Broth, 0.2% yeast extract, and 1.5% agar) plates, and colony counting was performed. The data are presented as the ratio of “intracellular live GAS at 4 h post-infection” to “total intracellular GAS at 2 h post-infection”, and “total intracellular GAS at 2 h post-infection” to “total attached GAS at 0.5 or 1 h post-infection”.

### Generation of knockout lines using CRISPR/Cas9

CRISPR/Cas9 [26] was used to knockout Beclin 1, Atg5, Atg7 or Bcl-xL as described previously [27]. CRISPR guide RNAs (gRNAs) were chosen that targeted an exon common to all splicing variants of the gene of interest (target sequence; Beclin 1, 5’- TCCAACAACAGCACCATGC-3’, Atg5, 5’-ATCAAGTTCAGCTCTTCCT-3’, Atg7, 5’-GCCCCTTTTAGTAGTGCCT-3’, Bcl-xL, 5’- AGACCCCCAGTGCCATCAA-3’). For CRISPR/Cas9 gene editing, HeLa cells were transfected with a gRNA-hyg vector containing the CRISPR target sequence and hCAS9 vector (Addgene 41815). Two days after transfection, untransfected cells were removed by selection with 300 μg/mL hygromycin B (Nacalai Tesque) and 750 μg/mL geneticin (G418) (Nacalai Tesque). Single colonies were expanded into 24-well plates, and depletion of the targeted gene was confirmed by immunoblotting. As a secondary screen of some knockout lines, genomic DNA was isolated from cells and the genomic regions were amplified using PCR. These PCR products were sequenced to confirm the presence of the desired frameshift insertions and deletions.

### Generation of stable cell lines

Stable cell lines were generated by retroviral expression as previously described [28]. Plat-E cells (kindly provided by T. Kitamura, The University of Tokyo) were transiently transfected using FuGENE HD Reagent with constructs based on pBABE-puro (Addgene, 1764) and cultured for 48 h. The resulting retrovirus containing supernatant was collected, and used to infect HeLa cells. Uninfected cells were removed by selection with 2 μg/mL puromycin (Invitrogen).

Additionally, for constructs based on pLenti6/V5-DEST, the virus was produced using the ViraPower lentiviral expression system (Invitrogen) according to the manufacturer’s protocol. Briefly, 293 FT cells were cotransfected using Lipofectamine 2000 (Invitrogen) with pLenti-mCherry-EmGFP-LC3 and the mixture of the packaging plasmids (Invitrogen), and cultured for 48 h. The viral supernatant was collected, and used to infect HeLa cells. After 24 h, uninfected cells were removed by selection on 5 μg/mL blasticidin (Invitrogen).

### Immunoprecipitation

Cells were harvested, washed with PBS, and lysed in a lysis buffer containing 50 mM Tris-HCl pH 7.5, 150 mM NaCl, 100 mM NaF, 10 mM EGTA, 1 mM Na_3_VO_4_, 5 μM ZnCl_2_, 10% glycerol, 1% Triton X-100, and proteinase inhibitor cocktail (Nacalai Tesque) for 30 min at 4°C. Lysates were then centrifuged, and obtained supernatants were pre-cleared by incubating with Protein G Sepharose 4B (GE Healthcare Life Sciences) for 1 h at 4°C. After brief centrifugation, the supernatants were reacted with anti-FLAG antibodies at 4°C overnight, and then Protein G Sepharose beads were added and allowed to react (with rotation) for 1 h at 4°C. Immunoprecipitates were collected by brief centrifugation, and the mixtures were washed 5 times with wash buffer containing 50 mM Tris-HCl pH 7.5, 150 mM NaCl, and 0.1% Triton X-100, and analyzed by immunoblotting.

### Statistical analysis

Co-localization and GcAV formation was quantified through direct visualization in a confocal microscope. Unless otherwise indicated, at least 50 GcAVs or 200 GAS-infected cells were counted per treatment in each experiment. At least three independent experiments were performed for each trial. Unless otherwise indicated, the mean ± standard deviation (SD) is shown. Data were tested by two-tailed Student’s *t*-test. *P* values less than 0.05 were considered to indicate statistical significance, and are marked * for *P* < 0.05, ** for *P* < 0.01, and n.s. for not significant.

## Results

### Effect of Bcl-xL on GAS internalization

Initially, to confirm the inhibitory function of Bcl-2 and Bcl-xL in starvation-induced autophagy, HeLa cells stably expressing green fluorescent protein-fused LC3 (GFP-LC3) were transfected with either empty FLAG control vector (FLAG-Control), FLAG-Bcl-2, or FLAG-Bcl-xL, and the number of lipid-conjugated forms of LC3 (LC3-II) puncta were counted under starvation conditions. LC3 puncta formation was inhibited in cells expressing both Bcl-2 and Bcl-xL under starvation conditions (S1A and S1B Fig). Notably, starvation-induced LC3 puncta formation was more suppressed in cells expressing Bcl-xL than those expressing Bcl-2. Similarly, western blotting analysis showed that the conversion ratio of LC3-II/LC3-I in both Bcl-2- and Bcl-xL-overexpressing cells was lower than that of cells transfected with control vector after a 2 h starvation period (S1C Fig). From these results, we could verify the inhibitory function of Bcl-2 and Bcl-xL in starvation-induced autophagy.

**Fig 1:**
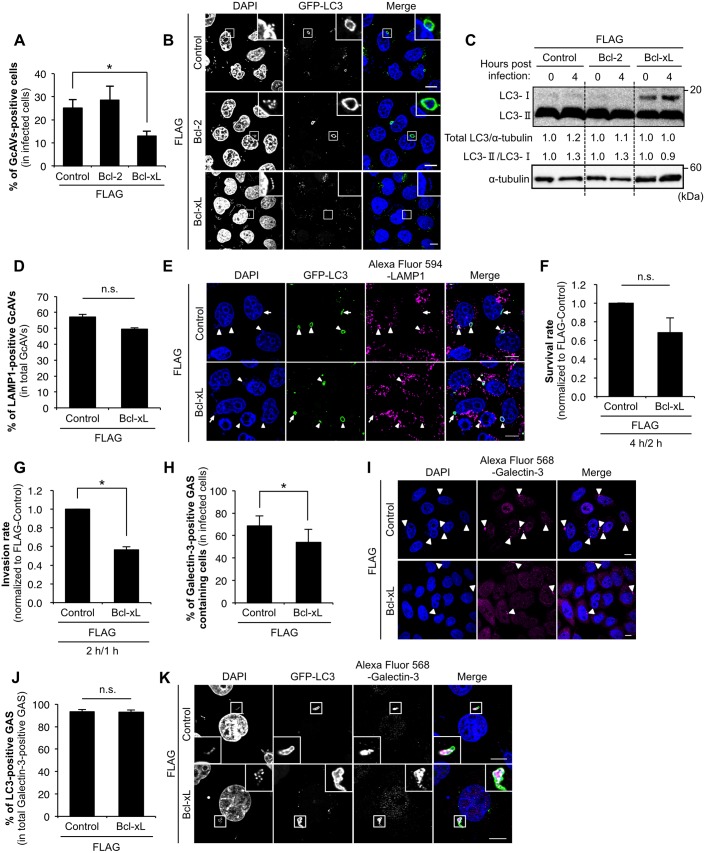
Bcl-xL suppresses GAS internalization. (A) The number of cells containing GcAV were counted and presented as the percentage of the total number of GAS-infected cells. HeLa cells stably expressing GFP-LC3 were transfected with FLAG-control, -Bcl-2, or -Bcl-xL and infected with GAS (MOI = 100) for 4 h. Cellular and bacterial DNA was stained with DAPI. The data shown represents result from >200 infected cells in terms of the mean value ± SD from three independent experiments. * *P* < 0.05. (B) Confocal microscopic images of GcAV in cells expressing either FLAG-control, -Bcl-2, or–Bcl-xL. Insets show expansion of the boxed areas. Scale bars, 10 μm. (C) The accumulation of LC3-II during GAS infection. HeLa cells were transfected with FLAG-control, -Bcl-2, or -Bcl-xL and infected with GAS (MOI = 100) for 4 h. Expression of LC3 was analyzed by western blotting using anti-LC3 antibody. (D) Co-localization efficiencies of GcAV and lysosomes were calculated as the percentage of the total number of GcAV. HeLa cells stably expressing GFP-LC3 were transfected with FLAG-control, or -Bcl-xL and infected with GAS (MOI = 100) for 4 h. Cells were then immunostained with an anti-LAMP1 antibody. Cellular and bacterial DNA was stained with DAPI. The data shown represent results of >50 GcAVs and each percentage represents the mean value ±SD from three independent experiments. (E) Confocal microscopic images of GcAV associated with lysosomes. White arrows show autophagosomes, white arrow heads show autolysosomes. Scale bars, 10 μm. (F) Intracellular survival rate of GAS. HeLa cells were transfected with FLAG-control, or -Bcl-xL, and infected with GAS (MOI = 100). After 1 h of infection, cells were washed with PBS and further incubated for 1 h with DMEM/10% FCS with gentamicin (100 μg/ml) to kill the extracellular bacteria. Cells were disrupted with distilled water and serial dilutions of cellular extracts were plated on THY agar plates, and colony counting was performed. The data presents the survival rate as the ratio of “intracellular live GAS at 4 h post-infection” to “total intracellular GAS at 2 h post-infection”. Data are representative of ≥ three independent experiments. (G) Invasion rate of GAS. HeLa Cells were transfected and infected as in (F). The data presents the invasion rate as the ratio of “total intracellular GAS at 2 h post-infection” to “total adherent GAS at 1 h post-infection”. Data are representative of ≥ three independent experiments. * *P* < 0.05. (H) The number of cells containing Galectin-3-positive GAS were counted and presented as the percentage of the total number of GAS-infected cells. HeLa cells stably expressing GFP-LC3 were transfected with either FLAG-control, -Bcl-2, or–Bcl-xL and infected with GAS (MOI = 100) for 4 h. Cells were then immunostained with an anti-Galectin-3 antibody. Cellular and bacterial DNA was stained with DAPI. The data shown represent results from >200 infected cells in terms of the mean value ± SD from three independent experiments. * *P* < 0.05. (I) Confocal microscopic images of Galectin-3-positive GAS. White arrow heads show Galectin-3-positive GAS. Scale bars, 10 μm. (J) Co-localization efficiencies of Galectin-3-positive GAS and GcAV were calculated as the percentage of the total number of Galectin-3-positive GAS. HeLa cells stably expressing GFP-LC3 were transfected with either FLAG-control, -Bcl-2, or–Bcl-xL, and infected with GAS (MOI = 100) for 4 h. Cells were then immunostained with an anti-Galectin-3 antibody. Cellular and bacterial DNA was stained with DAPI. The data shown represent results of >50 Galectin-3-positive GAS and each percentage represents the mean value ± SD from three independent experiments. * *P* < 0.05. (K) Confocal microscopic images of LC3-positive GAS in Galectin-3-positive GAS. Insets show expansion of the boxed areas. Scale bars, 10 μm.

Next, we assessed the role of Bcl-2 and Bcl-xL in GAS infection. Unlike under starvation conditions, the rate of GcAV formation was reduced in cells transfected with Bcl-xL, but not in those transfected with Bcl-2 (Fig 1A and 1B). Consistent with this, less accumulation of LC3-II was observed in cells transfected with Bcl-xL but not Bcl-2 during GAS infection (Fig 1C). On the other hand, the co-localization efficiency of LAMP1, a lysosomal membranes marker, with GcAV was not affected, and the intracellular survival rate of GAS was slightly decreased in Bcl-xL-overexpressing cells compared to that of the control vector-transfected cells (Fig 1D, 1E and 1F). To clarify the reason of decrease in GcAV formation in Bcl-xL-overexpressing cells, we evaluated the cell-internalization ability of GAS in control vector or Bcl-xL-overexpressing cells. Of note, overexpression of Bcl-xL resulted in the significant reduction (43%) in the number of invading GAS (Fig 1G). To further assess the correlation between internalization and autophagosome formation, we used Galectin-3, a marker of damaged endomembranes [29]. The percentage of Galectin-3-positive GAS in Bcl-xL-overexpressing cells decreased by 15% compared with that of the control vector-transfected cells (Fig 1H and 1I). However, LC3 recruitment to Galectin-3-poisitve GAS was not affected in Bcl-xL-overexpressing cells compared with the control vector-transfected cells (Fig 1J and 1K). Therefore, our data suggests that a decreased number of internalized GAS in Bcl-xL-overexpressing cells is responsible for the reduction in autophagic LC3 activity (GcAV formation and recruitment to endosomes), and that overexpression of Bcl-xL is not invariably involved in the fusion of GcAV with lysosomes, which eventually degrades GAS.

### Lack of Bcl-xL promotes GAS internalization and autophagosome-lysosome fusion

In Bcl-xL-overexpressing cells, Bcl-xL is suggested to regulate starvation-induced autophagy and internalization pathway of GAS. Next, we established a Bcl-xL KO HeLa cell line by genome editing (S2A and S2B Fig) and evaluated the role of endogenous Bcl-xL. Under starvation conditions, the number of LC3 puncta was increased approximately twofold in Bcl-xL KO cells compared to that of wild-type cells (S2C and S2D Fig). Interestingly, an increase of LC3 puncta was observed under not only starvation conditions but also nutrient-rich conditions. Consistent with this result, although the accumulation of LC3-II was observed in both cell-lines after a 2 h starvation period, the LC3-II levels of Bcl-xL KO cells under nutrient conditions seemed to be higher compared with that of wild-type cells (S2E Fig). These results suggest that endogenous Bcl-xL regulates either autophagosome formation or maturation, under both starvation and basal conditions.

**Fig 2:**
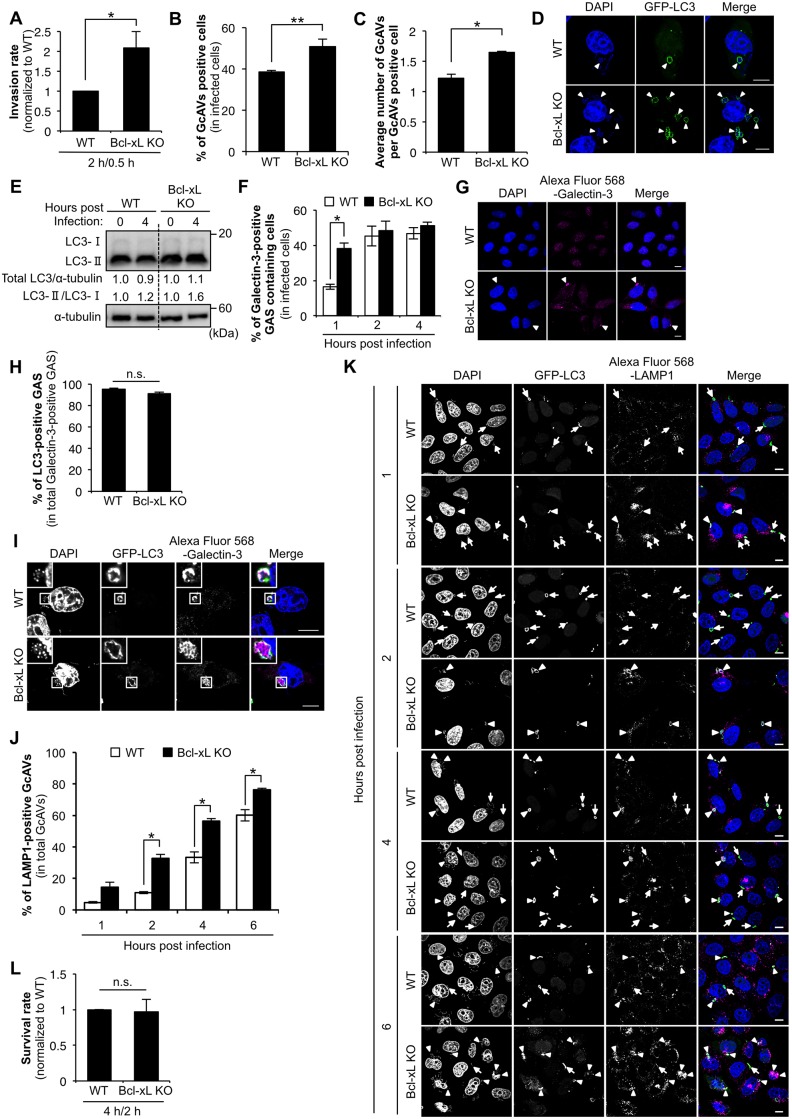
Endogenous Bcl-xL regulates GAS internalization and autophagosome-lysosome fusion during GAS infection. (A) Invasion rate of GAS in Bcl-xL KO cells. Both wild-type and Bcl-xL KO cells were infected with GAS (MOI = 100). At 0.5 h post-infection, cells were disrupted with distilled water and serial dilutions of cellular extracts were plated on THY agar plates, and colony counting was performed. The data presents the invasion rate as the ratio of “total intracellular GAS at 2 h post-infection” to “total adherent GAS at 0.5 h post-infection”. Data are representative of ≥ three independent experiments. * *P* < 0.05. (B) The number of cells containing GcAV were counted and presented as the percentage of the total number of GAS-infected cells. Wild-type and Bcl-xL KO cells stably expressing GFP-LC3 were infected with GAS (MOI = 100) for 4 h. Cellular and bacterial DNA was stained with DAPI. The data shown represent results from >200 infected cells in terms of the mean value ± SD from three independent experiments. ** *P* < 0.01. (C) Quantification of the number of GcAV per GcAV-positive cell. Wild-type and Bcl-xL KO cells stably expressing GFP-LC3 were infected and treated as in (B). The data shown represent results from >50 GcAV-positive cells in terms of the mean value ± SD from three independent experiments. * *P* < 0.05. (D) Confocal microscopic images of GcAV in Bcl-xL KO cells. White arrowheads show GcAV. Scale bars, 10 μm. (E) The accumulation of LC3-II during GAS infection. Wild-type and Bcl-xL KO cells were cultured and infected with GAS (MOI = 100) for 4 h. Expression of LC3 was analyzed by western blotting using anti-LC3 antibody. (F) The number of cells containing Galectin-3-positive GAS were counted and presented as the percentage of the total number of GAS-infected cells. Wild-type and Bcl-xL KO cells stably expressing GFP-LC3 were infected with GAS (MOI = 100) for indicated times. Cells were then immunostained with an anti-Galectin-3 antibody. Cellular and bacterial DNA was stained with DAPI. The data shown represent results from >200 infected cells in terms of the mean value ± SD from three independent experiments. * *P* < 0.05. (G) Confocal microscopic images of Galectin-3-positive GAS at 1 h post-infection. White arrow heads show Galectin-3-positive GAS. Scale bars, 10 μm. (H) Co-localization efficiencies of Galectin-3-positive GAS and GcAV were calculated as the percentage of total number of Galectin-3-positive GAS. Wild-type and Bcl-xL KO cells stably expressing GFP-LC3 were infected with GAS (MOI = 100) for 4 h. Cells were then immunostained with an anti-Galectin-3 antibody. Cellular and bacterial DNA was stained with DAPI. The data shown represent results of >50 Galectin-3-positive GAS and each percentage represents the mean value ± SD from three independent experiments. (I) Confocal microscopic images of LC3-positive GAS in Galectin-3-positive GAS in Bcl-xL KO cells. Insets show expansion of the boxed areas. Scale bars, 10 μm. (J) Co-localization efficiencies of GcAV and lysosomes in Bcl-xL KO cells. Wild-type and Bcl-xL KO cells stably expressing GFP-LC3 were infected with GAS (MOI = 100) for indicated times. Cells were then immunostained with an anti-LAMP1 antibody. Cellular and bacterial DNA was stained with DAPI. Co-localization efficiencies of GcAV and LAMP1 were calculated as the percentage of total number of GcAV. The data shown represent results of >50 GcAVs and each percentage represents the mean value ± SD from three independent experiments. * *P* < 0.05. (K) Confocal microscopic images of GcAV associated with lysosomes in Bcl-xL KO cells. White arrows show autophagosomes, white arrow heads show autolysosomes. Scale bars, 10 μm. (L) Intracellular survival rate of GAS in Bcl-xL KO cells. Both wild-type and Bcl-xL KO cells were infected and treated as in (A). The data presents the survival rate as the ratio of “intracellular live GAS at 4 h post-infection” to “total intracellular GAS at 2 h post-infection”. Data are representative of ≥three independent experiments.

Next, we assessed the influence of Bcl-xL on cell internalization and autophagy in GAS infection. The internalization rate of GAS in Bcl-xL KO cells increased approximately twofold in comparison with wild-type cells after infection (Fig 2A). The efficiency of formation of GcAV-positive cells in Bcl-xL KO cells was approximately 1.35-fold higher during infection compared with that of the wild-type cells (Fig 2B). In addition, the number of GcAVs per cell in Bcl-xL KO cells increased approximately 1.34-fold compared with that of the wild-type cells (Fig 2C and 2D). Similarly, LC-II levels in Bcl-xL KO cells were higher than that in wild-type cells during GAS infection (Fig 2E). The percentage of Galectin-3-positive GAS in Bcl-xL KO cells was about 2.32-fold higher than that in wild-type cells at 1 h post-infection, while there was no difference between wild-type and Bcl-xL KO cells at 2 and 4 h post-infection (Fig 2F and 2G). On the other hand, LC3 recruitment to Galectin-3-poisitve GAS was not different between wild-type and Bcl-xL KO cells (Fig 2H and 2I). These results indicate that endogenous Bcl-xL indirectly regulates GcAV formation by inhibiting GAS internalization, but does not affect LC3 recruitment to damaged GAS-containing endosomes. Next, to examine the influence of Bcl-xL on the autophagosome-lysosome fusion, we observed co-localization of LAMP1 with GcAV at 1, 2, 4 and 6 hours post infection. The rates of LAMP1-positive GcAV in Bcl-xL KO cells were higher than those in wild-type cells at all time points of infection (Fig 2J and 2K), suggesting that the promotion of GcAV-lysosome fusion in Bcl-xL KO cells dose not only reflect the increased number of invaded GAS. In addition, no significant difference was observed, but the survival rate of GAS in Bcl-xL KO cells reduced by approximately 3% compared with that in wild-type cells (Fig 2L) despite an excessive increase in the number of invading GAS (Fig 2A). These results suggest that endogenous Bcl-xL suppresses GAS-induced autophagy by inhibiting not only the GAS internalization process indirectly but also the autophagosome-lysosome fusion.

### Knockout of Beclin 1 inhibits GAS internalization

Bcl-xL is known to interact with Beclin 1 (also confirmed in S3A Fig) and to reduce the pro-autophagy activity of Beclin 1-PI3KC3, a crucial complex for the nucleation of autophagosome formation under starvation conditions [10, 11]. We established Beclin 1 KO HeLa cells (S3B and S3C Fig) and initially evaluated the role of endogenous Beclin 1 under starvation conditions. We confirmed the attenuation of starvation-induced autophagic activity by showing the reduction in LC3 puncta formation and LC3-II levels (S3D, S3E and S3F Fig) in Beclin 1 KO cells. These results were not inconsistent with that of a previous study [30].

**Fig 3:**
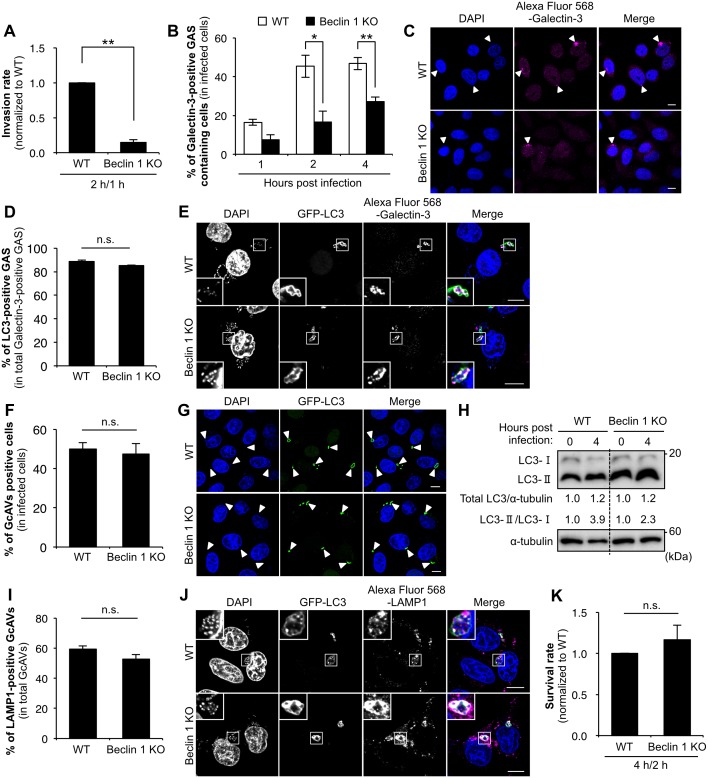
Knockout of Beclin 1 inhibits GAS internalization. (A) Invasion rate of GAS in Beclin 1 KO cells. Both wild-type and Beclin 1 KO cells were infected with GAS (MOI = 100). At 1 h post-infection, cells were disrupted with distilled water and serial dilutions of cellular extracts were plated on THY agar plates, and colony counting was performed. The data presents the invasion rate as the ratio of “total intracellular GAS at 2 h post-infection” to “total adherent GAS at 1 h post-infection”. Data are representative of ≥ three independent experiments. ** *P* < 0.01. (B) The number of cells containing Galectin-3-positive GAS were counted and presented as the percentage of the total number of GAS-infected cells. Wild-type and Beclin 1 KO cells stably expressing GFP-LC3 were infected with GAS (MOI = 100) for the indicated times. Cells were then immunostained with an anti-Galectin-3 antibody. Cellular and bacterial DNA was stained with DAPI. The data shown represent results from >200 infected cells in terms of the mean value ± SD from three independent experiments. * *P* < 0.05, ** *P* < 0.01. (C) Confocal microscopic images of Galectin-3-positive GAS at 4 h after infection. White arrow heads show Galectin-3-positive GAS. Scale bars, 10 μm. (D) Co-localization efficiencies of Galectin-3-positive GAS and GcAV were calculated as the percentage of total number of Galectin-3-positive GAS. Wild-type and Beclin 1 KO cells stably expressing GFP-LC3 were infected with GAS (MOI = 100) for 4 h. Cells were then immunostained with an anti-Galectin-3 antibody. Cellular and bacterial DNA was stained with DAPI. The data shown represent results of >50 Galectin-3-positive GAS and each percentage represents the mean value ± SD from three independent experiments. (E) Confocal microscopic images of LC3-positive GAS in Galectin-3-positive GAS in Beclin 1 KO cells. Insets show expansion of the boxed areas. Scale bars, 10 μm. (F) The number of cells containing GcAV were counted and presented as the percentage of the total number of GAS-infected cells. Wild-type and Beclin 1 KO cells stably expressing GFP-LC3 were infected with GAS (MOI = 100) for 4 h. Cellular and bacterial DNA was stained with DAPI. The data shown represent results from >200 infected cells in terms of the mean value ± SD from three independent experiments. (G) Confocal microscopic images of GcAV in Beclin 1 KO cells. White arrowheads show GcAVs. Scale bars, 10 μm. (H) The accumulation of LC3-II in Beclin 1 KO cells. Wild-type and Beclin 1 KO cells were infected with GAS (MOI = 100) for 4 h. Expression of LC3 was analyzed by western blotting using anti-LC3 antibody. (I) Co-localization efficiencies of GcAV and lysosomes in Beclin 1 KO cells. Wild-type and Beclin 1 KO cells stably expressing GFP-LC3 were infected with GAS (MOI = 100) for 4 h. Cells were then immunostained with an anti-LAMP1 antibody. Cellular and bacterial DNA was stained with DAPI. Co-localization efficiencies of GcAV and LAMP1 were calculated as the percentage of total number of GcAV. The data shown represent results of >50 GcAVs and each percentage represents the mean value ± SD from three independent experiments. (J) Confocal microscopic images of GcAV associated with lysosomes in Beclin 1 KO cells. Insets show expansion of the boxed areas. Scale bars, 10 μm. (K) Survival rate of GAS in Beclin 1 KO cells. Both wild-type and Beclin 1 KO cells were infected and treated as in (A). The data presents the survival rate as the ratio of “intracellular live GAS at 4 h post-infection” to “total intracellular GAS at 2 h post-infection”. Data are representative of ≥ three independent experiments.

Beclin 1 is also suggested to be involved in endocytosis [31–35]. To examine whether the inhibitory function of Bcl-xL in the internalization process of GAS is mediated through the Beclin 1 activation, we evaluated the role of endogenous Beclin 1 under GAS infection. The internalization rate of GAS in Beclin 1 KO cells decreased by approximately one fifth in comparison with that of wild-type cells (Fig 3A). To further investigate the involvement of other autophagy essential components on GAS internalization, we examined the internalization ability of GAS in Atg5 and Atg7 KO cells established by CRISPR/Cas9 system (S3G Fig). There was no significant difference in the GAS internalization between wild-type, Atg5 and Atg7 KO cells (S3H Fig), suggesting that the internalization defect of GAS in Beclin 1 KO cells is not a result of defect of autophagy process. In addition, the LC3 recruitment to damaged endosomes containing GAS (Galectin-3-poisitve GAS) was suppressed in Beclin 1 KO cells at 2 and 4 h post-infection in comparison with that of wild-type cells (Fig 3B and 3C). However, LC3 recruitment to Galectin-3-poisitve GAS was not different between wild-type and Beclin 1 KO cells (Fig 3D and 3E). From these results, we expected that a decrease in the number of invading GAS in Beclin 1 KO cells might subsequently result in downregulation of both GcAV formation and LC3-II levels. However, deletion of Beclin 1 did not affect GcAV formation and LC3-II levels (Fig 3F, 3G and 3H). In addition, the rate of LAMP1-positive GcAV and the survival rate of intracellular GAS were almost the same between wild-type and Beclin 1 KO cells (Fig 3I, 3J and 3K). Taken together, these results suggest that endogenous Beclin 1 partially phonocopies the defective internalization process of GAS observed in Bcl-xL-overexpressing cells, and that Beclin 1 is dispensable for Bcl-xL-mediated regulation of GcAV formation and GcAV-lysosome fusion.

### Bcl-xL-Beclin 1-UVRAG regulates GAS internalization

**Fig 4:**
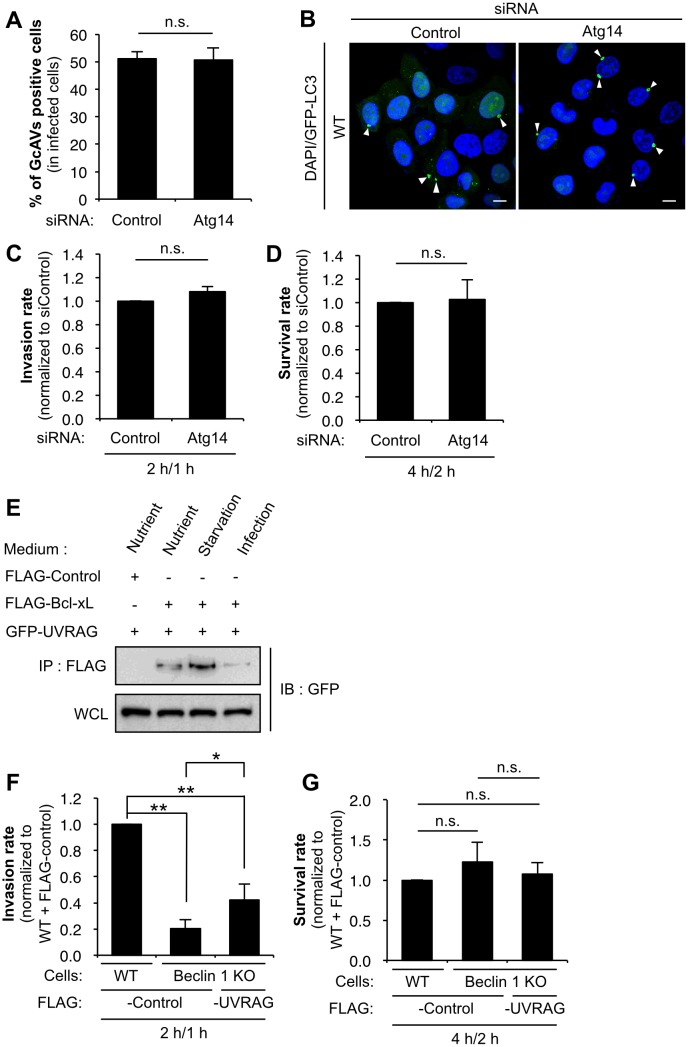
UVRAG but not Atg14 regulates GAS internalization. (A) The number of cells containing GcAV were counted and presented as the percentage of the total number of GAS-infected cells. HeLa cells stably expressing GFP-LC3 were transfected with a control siRNA or Atg14 siRNA and infected with GAS (MOI = 100) for 4 h. Cellular and bacterial DNA was stained with DAPI. The data shown represent results from >200 infected cells in terms of the mean value ± SD from three independent experiments. (B) Confocal microscopic images of GcAV in Atg14L knockdown cells. White arrowheads show GcAVs. Scale bars, 10 μm. (C) Invasion rate of GAS in Atg14L knockdown cells. HeLa cells were transfected with a control siRNA or Atg14 siRNA and infected with GAS (MOI = 100). At 1 h post-infection, cells were disrupted with distilled water and serial dilutions of cellular extracts were plated on THY agar plates, and colony counting was performed. The data presents the invasion rate as the ratio of “total intracellular GAS at 2 h post-infection” to “total adherent GAS at 1 h post-infection”. Data are representative of ≥ three independent experiments. (D) Intracellular survival rate of GAS in Atg14L knockdown cells. HeLa cells were transfected and infected as in (C). The data presents the survival rate as the ratio of “intracellular live GAS at 4 h post-infection” to “total intracellular GAS at 2 h post-infection”. Data are representative of ≥ three independent experiments. (E) UVRAG interacts with Bcl-xL. HEK293T cells transfected with FLAG-control or -Bcl-xL and EmGFP-UVRAG and cultured under nutrient-rich and starvation conditions for 2 h, or were infected with GAS for 4 h, and then subjected to immunoprecipitations with an anti-FLAG antibody. The immunoprecipitated proteins and total cell lysates were analyzed by immunoblotting with anti-GFP antibody. (F) Invasion rate of GAS in UVRAG-overexpressing Beclin 1 KO cells. Wild-type HeLa cells transfected with FLAG-control and Beclin 1 KO cells transfected with FLAG-control or FLAG-UVRAG were infected with GAS (MOI = 100). At 1 h post-infection, cells were disrupted with distilled water and serial dilutions of cellular extracts were plated on THY agar plates, and colony counting was performed. The data presents the invasion rate as the ratio of “total intracellular GAS at 2 h post-infection” to “total adherent GAS at 1 h post-infection”. Data are representative of ≥ three independent experiments. * *P* < 0.05. ** *P* < 0.01. (G) Intracellular survival rate of GAS in UVRAG-overexpressing Beclin 1 KO cells. Wild-type HeLa cells and Beclin 1 KO cells were transfected and infected as in (F). The data presents the survival rate as the ratio of “intracellular live GAS at 4 h post-infection” to “total intracellular GAS at 2 h post-infection”. Data are representative of ≥ three independent experiments.

Finally, we investigated the involvement of Atg14L and UVRAG in the internalization process and autophagy during GAS infection, because both proteins are shown to positively stimulate starvation-induced autophagy [18–21] and also to regulate the endocytic process through their interaction with Beclin 1 [22–25, 36]. In Atg14L knockdown cells (S4A Fig), LC3 puncta formation was suppressed under starvation conditions (S4B and S4C Fig), but GcAV formation was not affected during GAS infection (Fig 4A and 4B). In addition, both the internalization and intracellular survival rate of GAS in Atg14L knockdown cells was almost the same as those of the control cells (Fig 4C and 4D). These results suggest that Atg14L is required for LC3 formation during starvation, but is not involved in the internalization process and GcAV formation during GAS infection. Immunoprecipitation assay showed the direct interaction of UVRAG with Bcl-xL (Fig 4E). A decrease in the number of invading GAS in Beclin 1 KO cells was partially rescued by overexpression of UVRAG (Fig 4F and 4G), suggesting that Bcl-xL-Beclin 1-UVRAG regulates GAS internalization.

## Discussion

Intracellular infection provides a nutritionally rich environment, in which bacterial pathogens are protected from various elements of the host immune system including phagocytes, antibacterial peptides, humoral antibodies, and antibiotics [37]. Although, in many cases, cell-invading bacterial pathogens are efficiently eliminated by host defense mechanisms with phagosomes, GAS can survive either within phagosomes of phagocytic cells or within endosomes of epithelial cells [1]. While the beneficial effects of endocytosis-mediated internalization for GAS have been shown, host cells have developed several molecular mechanisms to eliminate the invading GAS such as bactericidal ROS generation, autophagic degradation, and apoptosis for removing infected cells by programmed suicide [2, 3, 5].

The host cell internalization of GAS is mediated by its surface proteins such as M proteins and fibronectin-binding proteins [38, 39], both of which bind fibronectin and activate PI3K. The activation of PI3K catalyzes phosphorylation of membrane-associated phosphatidylinositol, which binds to downstream targets including integrin-linked kinase (ILK) [40]. ILK can indirectly activate the small GTPases Rac, and Cdc42, which in turn can regulate actin cytoskeleton rearrangement and the internalization of GAS into endosomes [41]. The Bcl-2/Bcl-xL-Beclin 1-PI3K complex, a regulator of starvation-induced autophagy, is also considered to be involved in other vesicle trafficking process such as endocytosis, however, its role in GAS internalization and anti-bacterial autophagy remains unclear.

It has been clearly demonstrated that the anti-apoptotic Bcl-2 family members Bcl-2 and Bcl-xL can inhibit autophagy by binding to Beclin 1 and blocking its function [11, 42, 43]. In this study, we also confirmed the inhibitory function of both Bcl-2 and Bcl-xL in starvation-induced autophagosome formation. On the other hand, in GAS infection, we showed that Bcl-xL but not Bcl-2 regulated GcAV formation and that it is attributed to the number of internalized GAS. In addition, Bcl-xL also regulated GcAV-lysosome fusion, which consequently affects the intracellular survival of GAS. Overall, these results suggest that Bcl-xL inhibits GAS-induced autophagy directly by inhibiting autophagosome-lysosome fusion and indirectly by suppressing the GAS internalization. However, from our results, another question arises: why is Bcl-xL but not Bcl-2 related to the internalization process and autophagy during GAS infection? In this regard, previous studies have shown that Bcl-2 at the ER but not at the mitochondria inhibits starvation-induced autophagy [11], because production of PtdIns3P by ER-localized PI3KC3 may become the initiation signal for autophagosome formation [17]. On the other hand, Bcl-xL can also localize to both mitochondria and the ER, and it associates with Beclin 1 within mitochondria via its BH3 domain [44]. Although further studies are warranted to determine the activation site of Bcl-xL and/or the binding site of Bcl-xL-Beclin 1 during GAS infection, our data indicate that Bcl-xL may have a differential role among starvation-induced autophagy, GAS internalization, and GAS-induced autophagy as compared with Bcl-2.

Many studies have shown a central role for Beclin 1 in regulating autophagy [32, 45, 46], but its distinct role in other vesicle trafficking including endocytosis is not fully understood. There are some arguments for and against the endocytic role of Beclin 1 [31–35]. In addition, the direct participation of Beclin 1 in the regulation of autophagy and endocytosis-mediated internalization during bacterial infection has not been elucidated. Furthermore, as Bcl-xL regulated the internalization events in GAS infected cells, we assumed that Beclin 1, as a binding partner of Bcl-xL and a central component of the PI3KC3 complex should also mediate such processes. In Beclin 1 KO cells, a significant inhibition of autophagosome (LC3 puncta) formation and a slight decrease in LC3-II levels were observed under starvation conditions, which is mostly consistent with previous studies showing Beclin 1 is required for the recruitment of lipidated LC3 to autophagosomes but not invariably for LC3 lipidation [47]. By contrast, knockout of Beclin 1 resulted in a reduction in the number of internalized GAS. On the other hand, the rate of GcAV formation and the co-localization efficiency of lysosomes with GcAVs in Beclin 1 KO cells were almost same as that of wild-type cells. Therefore, Beclin 1 might function as an important regulator of GAS internalization with Bcl-xL, but it is dispensable for the Bcl-xL-mediated regulation of GAS-induced autophagy. Further studies are warranted to determine molecules regulating anti-bacterial autophagy with Bcl-xL.

Finally, we showed that the function of Beclin 1 in the GAS internalization process depended on its interaction with UVRAG, but not with Atg14L. In mammalian cells, Atg14L is required to recruit PI3KC3 to the formation site of autophagosomes [48]. Atg14L is traditionally considered to be an autophagy specific factor, but a recent study shows that it may be involved in the regulation of endocytosis [36]. Despite a clear contribution of Atg14L in autophagosome formation under starvation conditions, knockdown of Atg14L did not affect GcAV formation or the internalization of GAS. In contrast, defective internalization of GAS in Beclin 1 KO cells was partially rescued by overexpression of UVRAG. Interestingly, UVRAG could directly associate with Bcl-xL as well as Beclin 1. It has been shown that UVRAG promotes endocytic trafficking [25, 49–51]. In addition, crystal structure analysis of Beclin 1 suggests that the coiled-coil domain interaction between Beclin 1 and UVRAG has a higher affinity than that of Beclin 1 and Atg14L under normal conditions [52]. Therefore, Beclin 1 may use distinct Beclin 1 complexes depending on intra- and extracellular environmental changes such as starvation and bacterial infection and Beclin 1-UVRAG but not Beclin 1-Atg14 functions in the internalization process of GAS.

In summary, our data show that anti-apoptotic Bcl-xL inhibits GAS-induced autophagy directly by inhibiting autophagosome-lysosome fusion and indirectly by suppressing the GAS internalization through its interaction with Beclin 1-UVRAG. Our study expands our view of the biological function of the Bcl-xL-Beclin 1-UVRAG complex in the bacterial cell internalization process and anti-bacterial autophagy, which may ultimately contribute to the kinetics of intracellular bacteria, and will allow us to explore ways to manipulate the functions for potentially therapeutic purposes in infectious diseases by invading bacteria.

## Supporting Information

S1 Fig Overexpression of Bcl-2 and Bcl-xL inhibits LC3 puncta formation under starvation conditions. (A) Quantification of LC3 puncta per cell. HeLa cells stably expressing GFP-LC3 were transfected with FLAG-control, -Bcl-2, or -Bcl-xL and cultured under starvation conditions for 2 h. Confocal microscopic images were taken from these cells and the number of LC3 puncta was determined. At least 50 cells were counted in terms of the mean value ± SD from 10 images. * *P* < 0.05. ** *P* < 0.01. (B) Confocal microscopic images of LC3 puncta in Bcl-2 or Bcl-xL-overexpressing cells. Scale bars, 10 μm. (C) The accumulation of LC3-II under nutrient-rich, and starvation conditions. HeLa cells expressing FLAG-control, -Bcl-2, or -Bcl-xL in either complete medium or HBSS starvation medium were cultured for 2 h. Expression of LC3 was analyzed by western blotting using anti-LC3 antibody.
(TIFF)

S2 Fig Basal and starvation-induced autophagy is activated in Bcl-xL KO cells. (A) Immunoblotting analysis of Bcl-xL KO HeLa cells. Wild-type and Bcl-xL KO cells were cultured under nutrient-rich conditions. Expression of Bcl-xL was analyzed by western blotting using anti-Bcl-xL antibody. (B) Sequences of the wild-type Bcl-xL locus and mutated allele of obtained Bcl-xL KO cells around the target locus. Red characters represent the target sequence and blue characters represent the PAM motif. Deleted nucleotides are indicated by hyphens. (C) Quantification of LC3 puncta per cell. Wild-type and Bcl-xL KO HeLa cells stably expressing GFP-LC3 were cultured under starvation conditions for 2 h. Confocal microscopic images were taken from these cells and the number of LC3 puncta was determined. At least 50 cells were counted in terms of the mean value ± SD from 10 images. ** *P* < 0.01. (D) Confocal microscopic images of LC3 puncta. Scale bars, 10 μm. (E) The accumulation of LC3-II under nutrient-rich, and starvation conditions. Wild-type and Bcl-xL KO cells were cultured in either complete medium or HBSS starvation medium were cultured for 2 h. Expression of LC3 was analyzed by western blotting using anti-LC3 antibody.
(TIFF)

S3 Fig The role of endogenous Beclin 1 in starvation-induced and GAS-induced autophagosome formation. (A) Beclin 1 interacts with Bcl-xL under nutrient-rich and starvation conditions, and during GAS infection. HEK293T cells transfected with FLAG-control, -Bcl-2, or -Bcl-xL together with EmGFP-Beclin 1 were cultured under nutrient-rich and starvation conditions for 2 h, or were infected with GAS for 4 h, and then subjected to immunoprecipitation with an anti-FLAG antibody. The immunoprecipitated proteins and total cell lysates were analyzed by immunoblotting with anti-FLAG and anti-GFP antibodies. (B) Immunoblotting analysis of Beclin 1 KO HeLa cells. Wild-type and Beclin 1 KO cells were cultured under nutrient-rich conditions. Expression of Beclin 1 was analyzed by western blotting using anti-Beclin 1 antibody. (C) Sequences of the wild-type Beclin 1 locus and mutated allele of obtained Beclin 1 KO cells around the target locus. Red characters represent the target sequence and blue characters represent the PAM motif. Deleted nucleotides are indicated by hyphens. (D) Quantification of LC3 puncta per cell. Wild-type and Beclin 1 KO HeLa cells stably expressing GFP-LC3 were cultured under starvation conditions for 2 h. Confocal microscopic images were taken from these cells and the number of LC3 puncta was determined. At least 50 cells were counted in terms of the mean value ± SD from 10 images. * *P* < 0.05. (E) Confocal microscopic images of LC3 puncta in Beclin 1 KO cells. Scale bars, 10 μm. (F) The accumulation of LC3-II in Beclin 1 KO cells. Wild-type and Beclin 1 KO cells in either complete medium or HBSS starvation medium were cultured for 2 h. Expression of LC3 was analyzed by western blotting using anti-LC3 antibody. (G) Immunoblotting analysis of Atg5 and Atg7 KO HeLa cells. Wild-type and Atg5 and Atg7 KO cells were cultured under nutrient-rich conditions. Expressions of Atg5 and Atg7 were analyzed by western blotting using anti-Atg5 and Atg7 antibody. (H) Invasion rate of GAS in Atg5 KO and Atg7 KO cells. Wild-type, Atg5 KO and Atg7 KO cells were infected with GAS (MOI = 100). At 0.5 or 1 h post-infection, cells were disrupted with distilled water and serial dilutions of cellular extracts were plated on THY agar plates, and colony counting was performed. The data presents the invasion rate as the ratio of “total intracellular GAS at 2 h post-infection” to “total adherent GAS at 0.5 or 1 h post-infection”. Data are representative of ≥ three independent experiments. * *P* < 0.05.
(TIFF)

S4 Fig Atg14 regulates starvation-induced autophagosome formation. (A) Immunoblotting analysis of Atg14 knockdown HeLa cells. HeLa cells were transfected with either control siRNA or Atg14 siRNA. Expression of Atg14 was analyzed by western blotting using anti-Atg14 antibody. (B) Quantification of LC3 puncta per cell. HeLa cells stably expressing GFP-LC3 were transfected with a control siRNA or Atg14 siRNA and cultured under starvation conditions for 2 h. Confocal microscopic images were taken from these cells and the number of LC3 puncta were determined. At least 50 cells were counted in terms of the mean value ± SD from 10 images. ** *P* < 0.01. (C) Confocal microscopic images of LC3 puncta in Atg14K knockdown cells. Scale bars, 10 μm.
(TIFF)
