# Supplementary figures and images for: Bcl-xL Affects Group A Streptococcus-Induced Autophagy Directly, by Inhibiting Fusion between Autophagosomes and Lysosomes, and Indirectly, by Inhibiting Bacterial Internalization via Interaction with Beclin 1-UVRAG
Source: PLoS One. 2017 Jan 13;12(1):e0170138. doi: 10.1371/journal.pone.0170138 (PMC5235370; doi:10.1371/journal.pone.0170138)

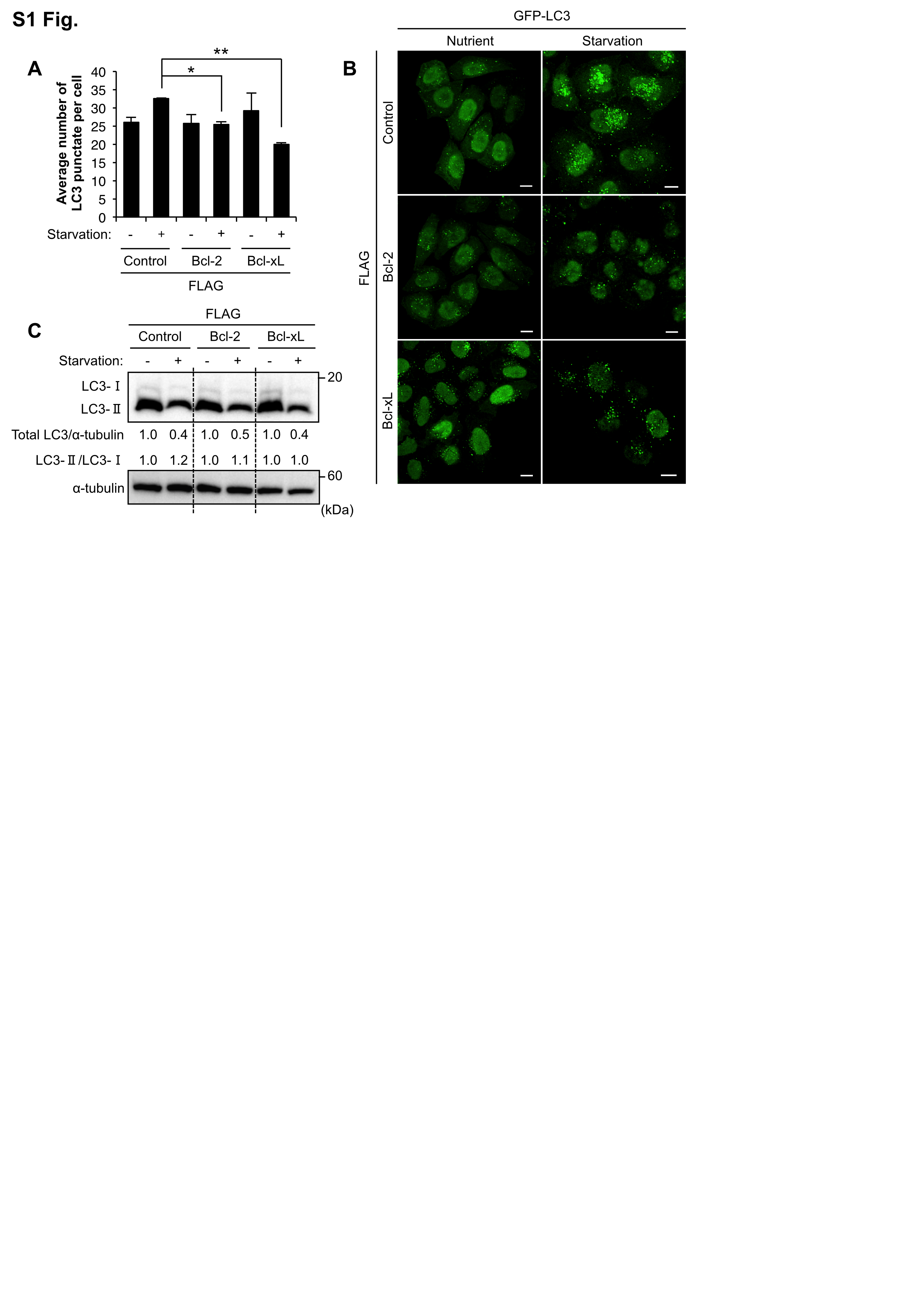

Supplement: S1 Fig — (A) Quantification of LC3 puncta per cell. HeLa cells stably expressing GFP-LC3 were transfected with FLAG-control, -Bcl-2, or -Bcl-xL and cultured under starvation conditions for 2 h. Confocal microscopic images were taken from these cells and the number of LC3 puncta was determined. At least 50 cells were counted in terms of the mean value ± SD from 10 images. * P < 0.05. ** P < 0.01. (B) Confocal microscopic images of LC3 puncta in Bcl-2 or Bcl-xL-overexpressing cells. Scale bars, 10 μm. (C) The accumulation of LC3-II under nutrient-rich, and starvation conditions. HeLa cells expressing FLAG-control, -Bcl-2, or -Bcl-xL in either complete medium or HBSS starvation medium were cultured for 2 h. Expression of LC3 was analyzed by western blotting using anti-LC3 antibody. (TIFF) [file pone.0170138.s001.tiff]

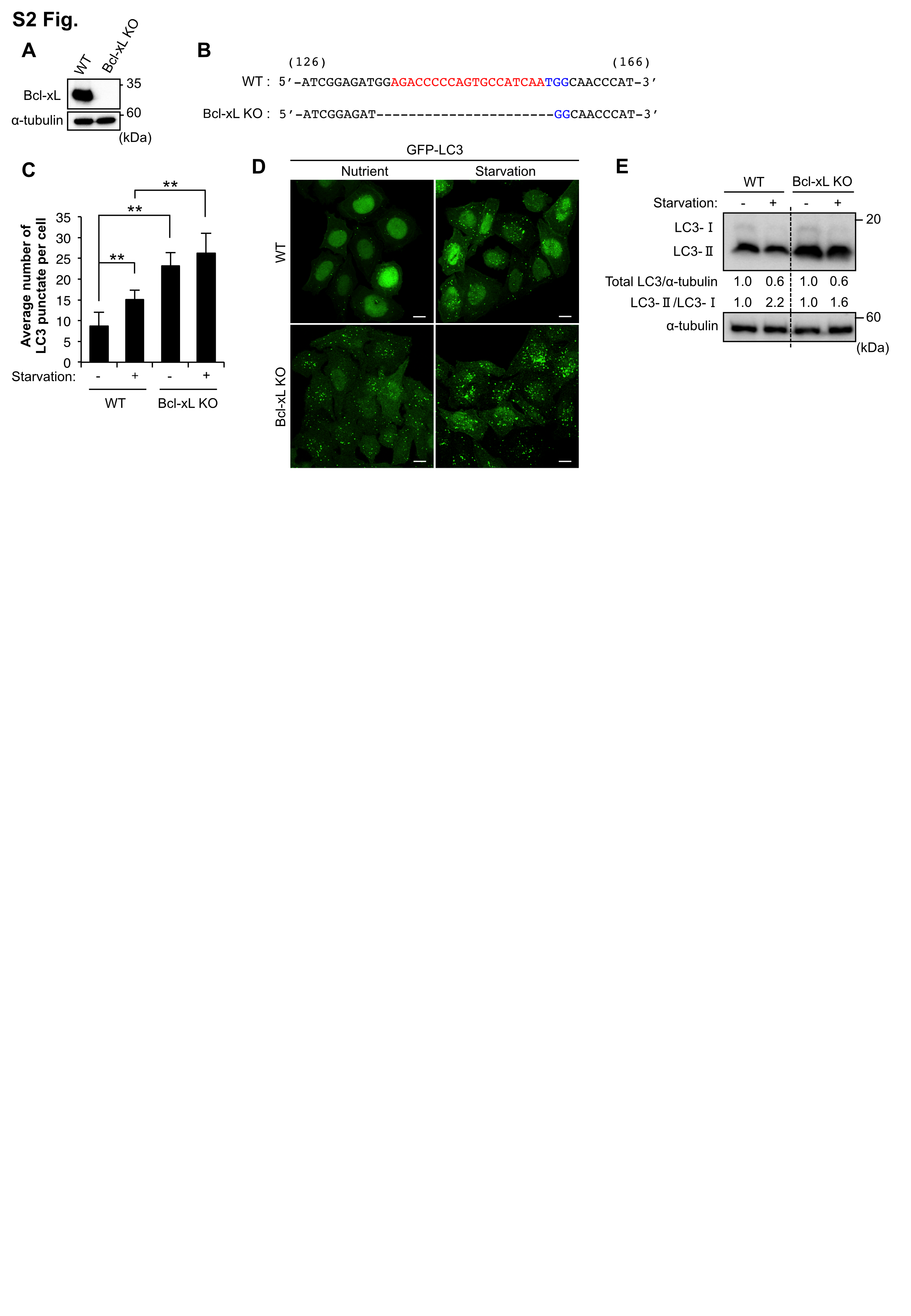

Supplement: S2 Fig — (A) Immunoblotting analysis of Bcl-xL KO HeLa cells. Wild-type and Bcl-xL KO cells were cultured under nutrient-rich conditions. Expression of Bcl-xL was analyzed by western blotting using anti-Bcl-xL antibody. (B) Sequences of the wild-type Bcl-xL locus and mutated allele of obtained Bcl-xL KO cells around the target locus. Red characters represent the target sequence and blue characters represent the PAM motif. Deleted nucleotides are indicated by hyphens. (C) Quantification of LC3 puncta per cell. Wild-type and Bcl-xL KO HeLa cells stably expressing GFP-LC3 were cultured under starvation conditions for 2 h. Confocal microscopic images were taken from these cells and the number of LC3 puncta was determined. At least 50 cells were counted in terms of the mean value ± SD from 10 images. ** P < 0.01. (D) Confocal microscopic images of LC3 puncta. Scale bars, 10 μm. (E) The accumulation of LC3-II under nutrient-rich, and starvation conditions. Wild-type and Bcl-xL KO cells were cultured in either complete medium or HBSS starvation medium were cultured for 2 h. Expression of LC3 was analyzed by western blotting using anti-LC3 antibody. (TIFF) [file pone.0170138.s002.tiff]

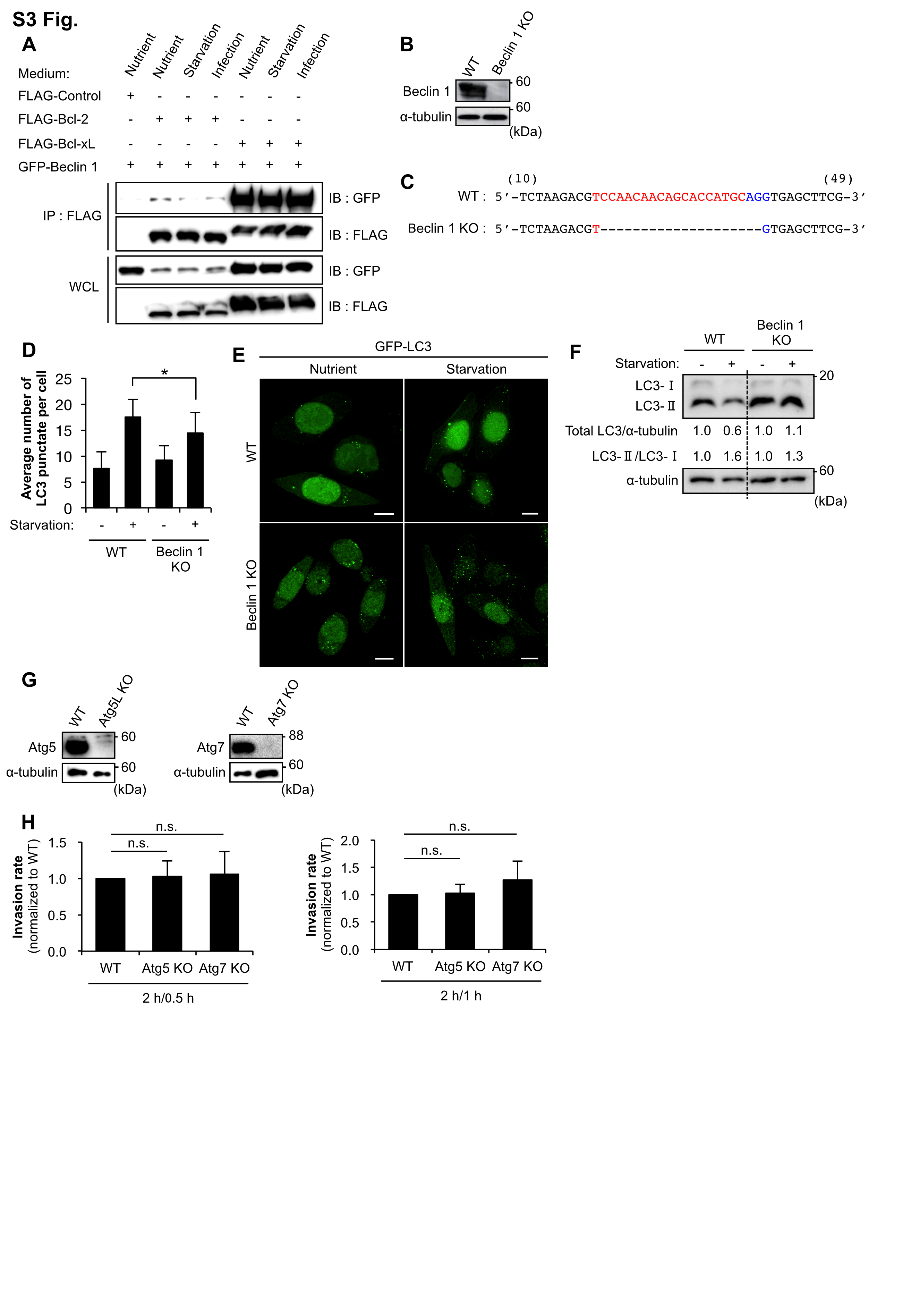

Supplement: S3 Fig — (A) Beclin 1 interacts with Bcl-xL under nutrient-rich and starvation conditions, and during GAS infection. HEK293T cells transfected with FLAG-control, -Bcl-2, or -Bcl-xL together with EmGFP-Beclin 1 were cultured under nutrient-rich and starvation conditions for 2 h, or were infected with GAS for 4 h, and then subjected to immunoprecipitation with an anti-FLAG antibody. The immunoprecipitated proteins and total cell lysates were analyzed by immunoblotting with anti-FLAG and anti-GFP antibodies. (B) Immunoblotting analysis of Beclin 1 KO HeLa cells. Wild-type and Beclin 1 KO cells were cultured under nutrient-rich conditions. Expression of Beclin 1 was analyzed by western blotting using anti-Beclin 1 antibody. (C) Sequences of the wild-type Beclin 1 locus and mutated allele of obtained Beclin 1 KO cells around the target locus. Red characters represent the target sequence and blue characters represent the PAM motif. Deleted nucleotides are indicated by hyphens. (D) Quantification of LC3 puncta per cell. Wild-type and Beclin 1 KO HeLa cells stably expressing GFP-LC3 were cultured under starvation conditions for 2 h. Confocal microscopic images were taken from these cells and the number of LC3 puncta was determined. At least 50 cells were counted in terms of the mean value ± SD from 10 images. * P < 0.05. (E) Confocal microscopic images of LC3 puncta in Beclin 1 KO cells. Scale bars, 10 μm. (F) The accumulation of LC3-II in Beclin 1 KO cells. Wild-type and Beclin 1 KO cells in either complete medium or HBSS starvation medium were cultured for 2 h. Expression of LC3 was analyzed by western blotting using anti-LC3 antibody. (G) Immunoblotting analysis of Atg5 and Atg7 KO HeLa cells. Wild-type and Atg5 and Atg7 KO cells were cultured under nutrient-rich conditions. Expressions of Atg5 and Atg7 were analyzed by western blotting using anti-Atg5 and Atg7 antibody. (H) Invasion rate of GAS in Atg5 KO and Atg7 KO cells. Wild-type, Atg5 KO and Atg7 KO cells were infected with [file pone.0170138.s003.tiff]

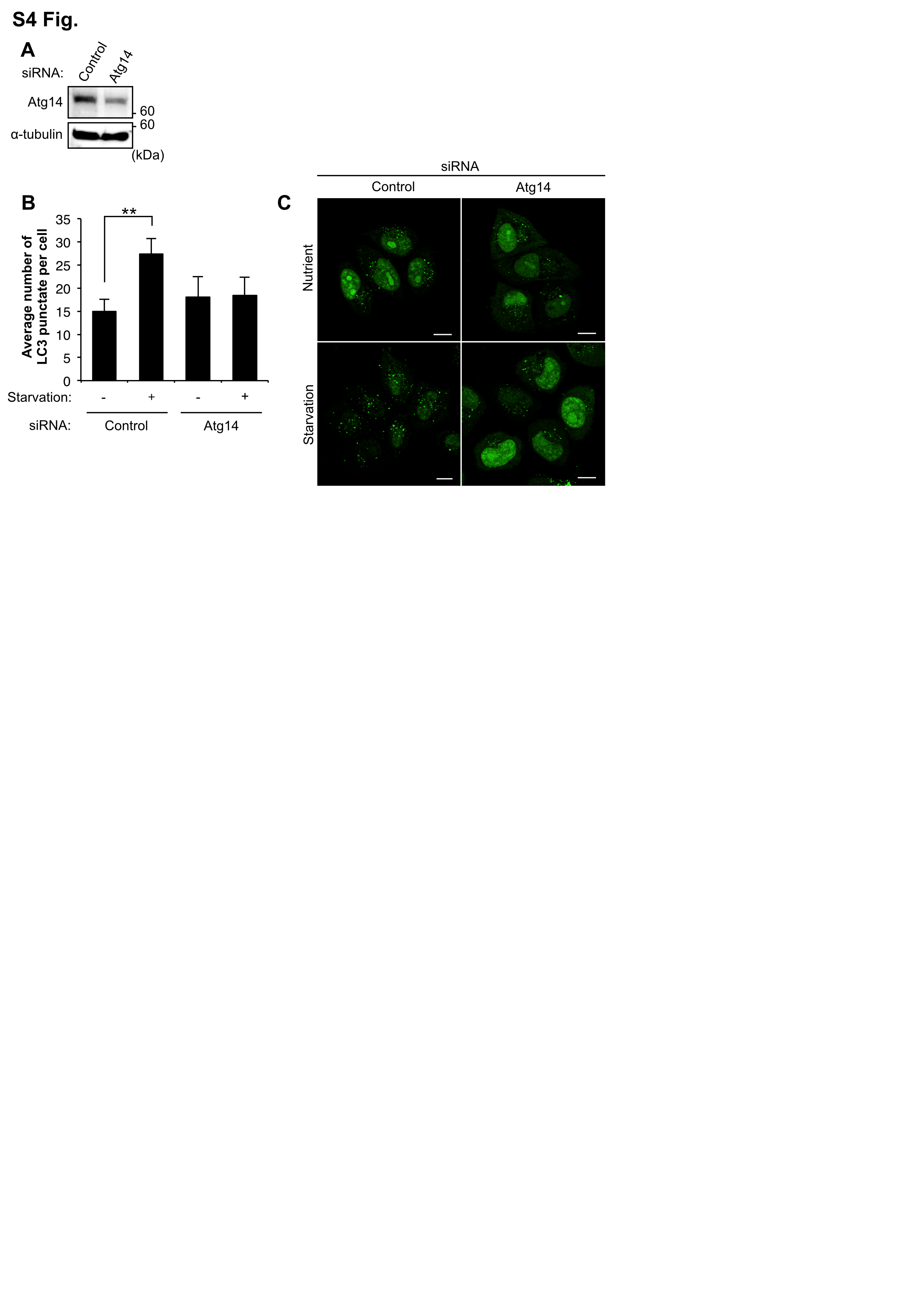

Supplement: S4 Fig — (A) Immunoblotting analysis of Atg14 knockdown HeLa cells. HeLa cells were transfected with either control siRNA or Atg14 siRNA. Expression of Atg14 was analyzed by western blotting using anti-Atg14 antibody. (B) Quantification of LC3 puncta per cell. HeLa cells stably expressing GFP-LC3 were transfected with a control siRNA or Atg14 siRNA and cultured under starvation conditions for 2 h. Confocal microscopic images were taken from these cells and the number of LC3 puncta were determined. At least 50 cells were counted in terms of the mean value ± SD from 10 images. ** P < 0.01. (C) Confocal microscopic images of LC3 puncta in Atg14K knockdown cells. Scale bars, 10 μm. (TIFF) [file pone.0170138.s004.tiff]
